# Noradrenaline depresses facial stimulation-evoked cerebellar MLI-PC synaptic transmission via α2-AR/PKA signaling cascade in vivo in mice

**DOI:** 10.1038/s41598-023-42975-5

**Published:** 2023-09-23

**Authors:** Jun-Ya Wang, Wen-Cai Weng, Ting-Qi Wang, Yue Liu, De-Lai Qiu, Mao-Cheng Wu, Chun-Ping Chu

**Affiliations:** 1 Department of Physiology and Pathophysiology, College of Medicine, Yanbian University, Yanji, China; 2 Department of Physiology, College of Basic Medicine, Jilin Medical University, Jilin, 132013 Jilin China; 3 Department of Osteology, Affiliated Hospital of Yanbian University, Yanji, 133002 Jilin China; 4 Department Radiology, Dalian Xinhua Hospital, Dalian University, Dalian, China

**Keywords:** Neurophysiology, Sensory processing

## Abstract

The noradrenergic fibers of the locus coeruleus, together with mossy fibers and climbing fibers, comprise the three types of cerebellar afferents that modulate the cerebellar neuronal circuit. We previously demonstrated that noradrenaline (NA) modulated synaptic transmission in the mouse cerebellar cortex via adrenergic receptors (ARs). In the present study, we investigated the effect of NA on facial stimulation-evoked cerebellar molecular layer interneuron (MLI)-Purkinje cell (PC) synaptic transmission in urethane-anesthetized mice using an in vivo cell-attached recording technique and a pharmacological method. MLI-PC synaptic transmission was induced by air-puff stimulation (duration: 60 ms) of the ipsilateral whisker pad, which exhibited positive components (P1 and P2) accompanied by a pause in simple spike activity. Cerebellar molecular layer application of NA (15 µM) decreased the amplitude and area under the curve of P1, and the pause in simple spike activity, but increased the P2/P1 ratio. The NA-induced decrease in P1 amplitude was concentration-dependent, and the half-inhibitory concentration was 10.94 µM. The NA-induced depression of facial stimulation-evoked MLI-PC GABAergic synaptic transmission was completely abolished by blockade of α-ARs or α2-ARs, but not by antagonism of α1-ARs or β-ARs. Bath application of an α2-AR agonist inhibited MLI-PC synaptic transmission and attenuated the effect of NA on the synaptic response. NA-induced depression of MLI-PC synaptic transmission was completely blocked by a mixture of α2A- and 2B-AR antagonists, and was abolished by inhibition of protein kinase A. In addition, electrical stimulation of the molecular layer evoked MLI-PC GABAergic synaptic transmission in the presence of an AMPA receptor antagonist, which was inhibited by NA through α2-ARs. Our results indicate that NA inhibits MLI-PC GABAergic synaptic transmission by reducing GABA release via an α2-AR/PKA signaling pathway.

## Introduction

The cerebellar cortex is composed of a molecular layer (ML), Purkinje cell (PC) layer, and granular cell layer, which mainly comprise PCs, molecular layer interneurons (MLIs), granule cells (GCs), and Golgi cells. Sensory information is conveyed to the cerebellar cortex by mossy fibers (MFs) and climbing fibers, which regulates the outputs of PCs. The PCs are core components of the cerebellar cortex, receiving projections from other cortical neurons and providing signals to the deep cerebellar nucleus^1^. MLIs inhibit PCs, which receive excitatory input from parallel fibers and inhibitory inputs from other interneurons^2–4^. Under in vivo conditions, sensory stimulation excites MLIs, which leads to the inhibition of PCs; this suggests that MLIs are critical for controlling PCs, which process sensory information in the mouse cerebellar cortex^5–8^.

Noradrenaline (NA) is an essential neurotransmitter and neuromodulator that has been widely investigated in the central nervous system. NAergic neurons in the locus coeruleus (LC) are the major source of NA in the mammalian brain, from which NAergic fibers project to all other parts of the brain, including the forebrain and cerebellum^9–11^. Anatomical studies indicate that NAergic fibers are distributed throughout the cerebellar cortex^12,13^. Monoamine fibers (including NAergic fibers), MFs, and climbing fibers are the three classes of cerebellar afferent inputs^14, 15^. The LC-NA system is considered to play an important modulatory role in sensory information processing and physiological processes downstream of the cerebellum, such as movement coordination and motor learning^16–18^.

Adrenergic receptors (ARs) are G protein-coupled receptors divided into α- (α1A, α1B, α1D, α2A, α2B, α2C), and β-receptor (β1–β3) types^19^. NA regulates synaptic transmission in cerebellar neurons by activating distinct AR subunits. Activation of α1- and α2-ARs inhibits parallel fiber-PC synaptic transmission, whereas activation of β-ARs enhances parallel fiber-PC synaptic transmission through the protein kinase A (PKA) signaling pathway^20–22^. In contrast, activation of β3-ARs depresses parallel-PC excitatory postsynaptic currents via the PI3K signaling pathway^23^. Additionally, NA enhances inhibitory neurotransmitter release through α1- and β-ARs, while also inhibiting the excitability of interneurons via the activation of α2-ARs^20, 24^. Under in vivo conditions, NA enhances inhibitory inputs of PCs by exciting MLIs, leading to the inhibition of PCs^25^. Moreover, NA reduces glutamate release at climbing fiber-PC synapses^26, 27^ and depresses facial stimulation-evoked MF-GC synaptic transmission via the activation of α2-ARs^28^.

NA modulates synaptic transmission in cerebellar cortical neurons, although the mechanism through which NA modulates sensory stimulation-evoked MLI-PC synaptic transmission in living animals remains unknown. Therefore, we investigated the effect of NA on facial stimulation-evoked MLI-PC synaptic transmission in urethane-anesthetized mice using in vivo electrophysiological and pharmacological approaches.

## Materials and methods

### Anesthesia and surgical procedures

Experimental procedures were approved by the Animal Care and Use Committee of Yanbian University and performed in accordance with the animal welfare guide lines of the National Institutes of Health. Permission No. is SYXK (Ji) 2011-006. Anesthesia and surgical procedures have been described previously^5, 6^. In brief, either male (n = 83) or female (n = 83) 6–8 weeks old ICR mice were anesthetized with urethane (1.3 g/kg body weight, intraperitoneal injection). Mice were tracheotomized to avoid respiratory obstruction, and fixed on a stereotaxic frame. A 1–1.5 mm craniotomy was opened to expose the cerebellar surface of Crus II. The surface of cerebellum was superfused with oxygenated artificial cerebrospinal fluid (ACSF: 125 mM NaCl, 3 mM KCl, 1 mM MgSO_4_, 2 mM CaCl_2_, 1 mM NaH_2_PO_4_, 25 mM NaHCO_3_, and 10 mM D-glucose) with a peristaltic pump (Gilson Minipulse 3; Villiers, LeBel, France) at 0.5 mL/min. Rectal temperature was monitored and maintained at 37.0 ± 0.2 °C using body temperature equipment.

### Cell-attached recordings and stimulation

The cell-attached recordings from PCs were performed with an Axopatch-700B amplifier (Molecular Devices, Foster City, CA, United States) under current clamp conditions (I = 0). The signals were acquired through a Digidata 1550 series analog-to-digital interface on a personal computer using Clampex 10.4 software (Molecular Devices). Recording electrodes were filled with ACSF, with resistances of 3–5 MΩ. Blind cell-attached recordings from PCs were performed at depths about 200 µM under the pia mater membrane. When neuronal spike firing appeared, micro-adjusting the electrode position to make the spike discharge amplitude moderate. Then, slowly release the positive pressure of the recording electrode and apply a slight negative pressure to format a cell-attached recording. PCs were identified by firing of regular spontaneous SS and irregular complex spikes.

**Figure 1:**
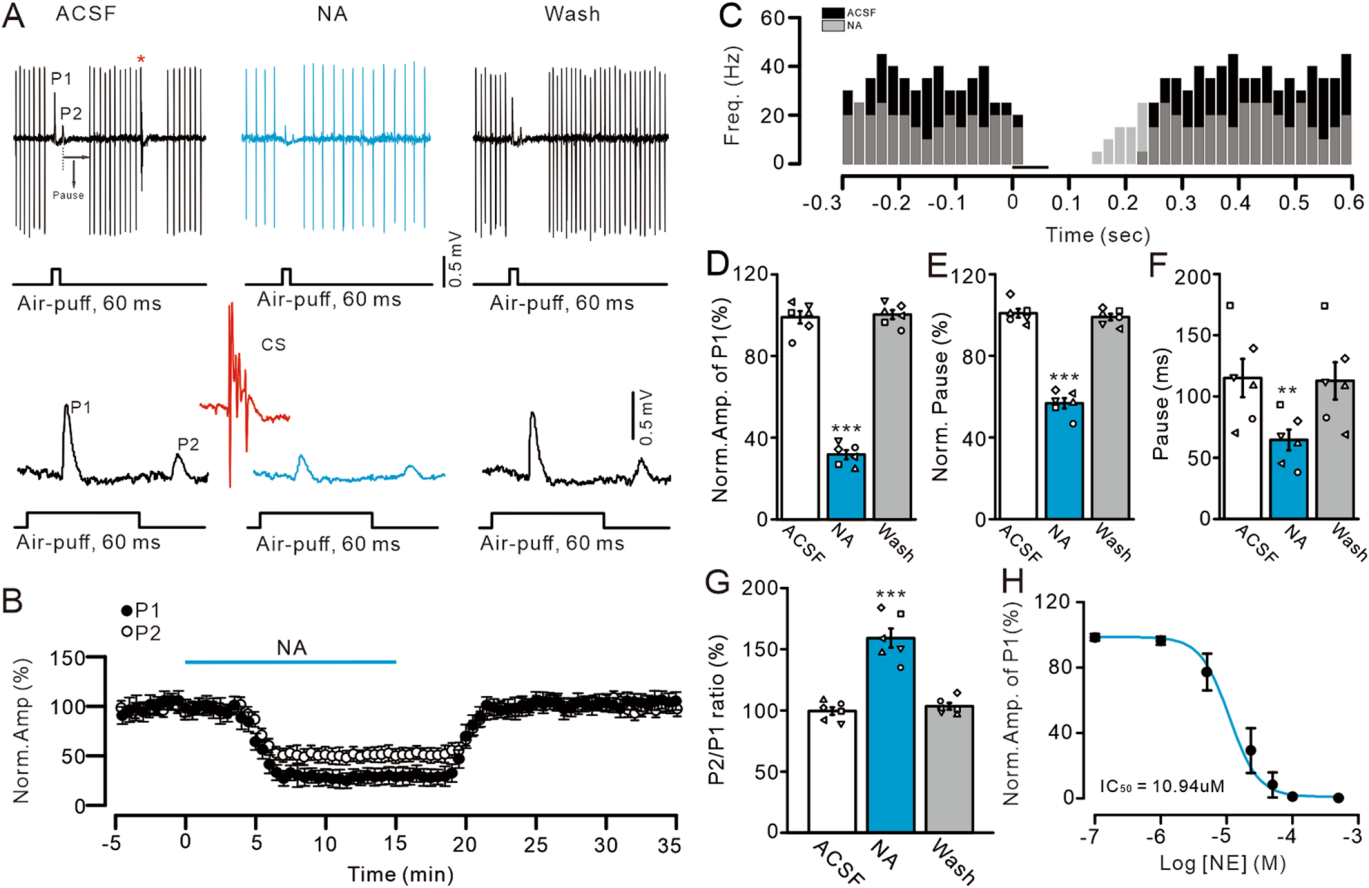
NA depresses facial stimulation-evoked MLI-PC synaptic transmission in vivo in mice. (**A**) Upper: representative cell-attached recording traces showing air-puff stimulation (60 ms; 60 psi)–evoked responses in a cerebellar PC during application of ACSF, NA (15 µM), and washout. Lower: enlarged traces of upper panel showing the facial stimulation-evoked MLI-PC synaptic transmission and the spontaneous complex spike marked with an asterisk. (**B**) Summary of data showing the time course of normalized amplitude of P1 and P2. (**C**) Instant frequency of the simple spike events showing a PC recorded in (**A**) in response to air-puff stimulus (black bar, 60 ms) in ACSF (black) and NA (15 µM; grey). (**D**) Bar graphs show the normalized amplitude of P1. (**E**–**G**) Bar graphs show the normalized pause of SS (**E**), the time of pause (**F**) and P2/P1 ratio (**G**). (**H**) The concentration–response curve showing the NA-induced decrease in amplitude of P1. The IC_50_ was 10. 94 µM. ***P < 0.001 vs. ACSF; **P < 0.01 vs. ACSF; n = 6 in each group.

The sensory stimulation was performed by air-puff (60 ms; 60 psi) of ipsilateral whisker pad through a 12-gauge stainless steel tube connected to a pressurized injection system (Picospritzer R III; Parker Hannifin Co, Pine Brook, Fairfield, NJ, United States). The air-puff stimulation was controlled by a personal computer, and was synchronized with the electrophysiological recordings via a Master 8 controller (A.M.P.I., Jerusalem, Israel) and Clampex 10.4 software. Air-puff stimulation (60 ms, 60 psi) of ipsilateral whisker pad induces MLI-PC synaptic responses, which expressed P1, P2 and a pause of SS firing in cerebellar PCs (Fig. 1A). According to our previous study, P1 and P2 were identified as MLI-PC synaptic induced by stimulation-on and -off, respectively^5^.

Electrical stimulation of molecular layer was performed by a stimulating electrode containing ACSF (0.1–0.5 MΩ). The stimulation electrode was placed under the cerebellar surface and close to the recorded PC. The current pulses (200 µs, 50–100 µA) at 30 s intervals were delivered through a glass electrode mounted on a remote-controlled micromanipulator (MP-385; Sutter Instrument Company, Novato, CA, USA) by an isolated stimulator (Isoflex; A.M.P.I., Jerusalem, Israel). The stimulator was synchronized with electrophysiology recording via a Master 8 controller (A.M.P.I.) and Clampex10.4 software (Molecular Device, Foster City, CA, USA). In order to recording the stimulation-evoked MLI-PC GABAergic synaptic response, an AMPA receptor antagonist, NBQX (50 µM) was added in ACSF to block glutamatergic excitatory synaptic transmission.

### Chemicals

The reagents included urethane; phentolamine (Phen), a nonselective α-AR antagonist; propranolol (Prop), a nonselective β-AR blocker; prazosin (Praz), an α1-AR antagonist; yohimbine (Yohim), an α2-AR antagonist, were bought from Sigma-Aldrich (Shanghai, China). NA; UK14304 (UK), an α2-AR agonist; BRL44408, an α2A-AR antagonist; Imiloxan, a highly selective α2B-AR antagonist; JP1302, a potent and selective α2C-AR antagonist; KT5720, a selective protein kinase A inhibitor; chelerythrine, a selective protein kinase C inhibitor; Gabazine (SR95531), a selective GABA_A_ receptor antagonist and NBQX (2,3-dioxo-6-nitro-1,2,3,4-tetrahydrobenzo[f] quinoxaline-7-sulfonamide), an AMPA receptor antagonist, were purchased from Tocris (Bristol, UK). All the drugs were finally dissolved in ACSF. In order to avoid the influence of NA on mossy fiber-granule cell synaptic transmission, NA was microinjected onto the molecular layer above the recorded PCs at 0.1 µL/s by a micropump (KDS-210, KD Scientific, Holliston, MA, United States). Molecular layer microapplication of NA inhibited the facial stimulation-evoked MLI-PC synaptic transmission, but did less affect the evoked mossy fiber-granule cell response (Supplemental Fig. 1). The other drugs were bath applied directly onto the cerebellar surface by a peristaltic pump (Gilson Mini pulse 3; Villiers-Le-Bel, France) at 0.5 mL/min. After a stable cell-attached recording was configured, the baseline was recorded, then perfusion of chemicals was done. In some experiments involving PKA or PKC inhibitors, application of KT5720 (1 µM) or chelerythrine (30 µM) was began at least 30 min before electrical recording and continuing throughout a experiment.

### Statistical analysis

**Figure 2:**
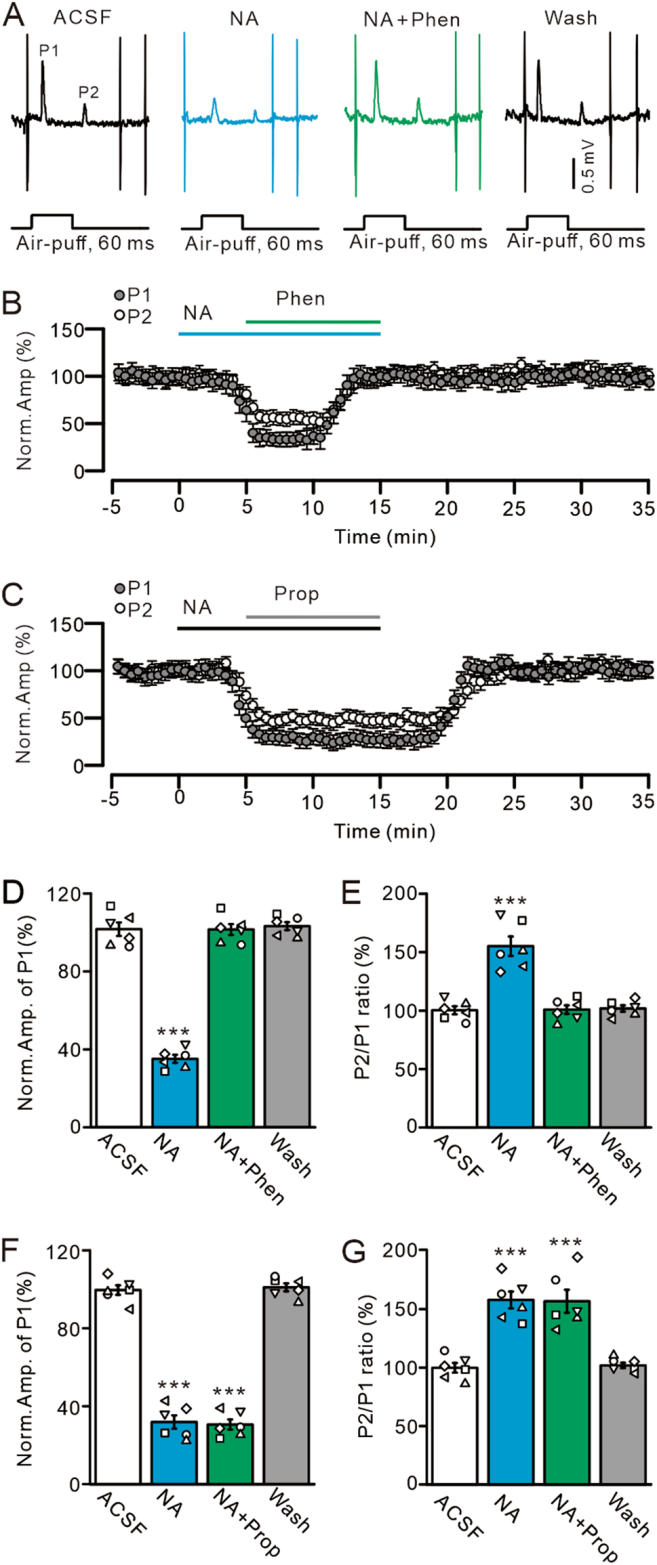
Blockade of α-AR abolishes NA-induced inhibition of the MLI-PC synaptic transmission. (**A**) Representative cell-attached recording traces showing air-puff stimulation (60 ms; 60 psi)-evoked responses in a cerebellar PC in treatments with ACSF, NA (15 µM), NA + phentolamine (Phen; 100 µM), and recovery (washout). (**B**) Summary of data showing the time course of normalized amplitude of P1 and P2 in treatments with ACSF, NA, NA + phentolamine, and recovery. (**C**) Summary of data showing the time course of normalized amplitude of P1 and P2 in treatments with ACSF, NA (15 µM), NA + propranolol (Prop; 100 µM) and washout. (**D**,**E**) Bar graphs show the normalized amplitude of P1 (**D**) and P2/P1 ratio (**E**) during treatments with ACSF, NA, NA + Phen and washout. (**F**,**G**) Summary of data show the normalized amplitude of P1 (**F**) and P2/P1 ratio (**G**) during treatments with ACSF, NA, NA + propranolol and washout. ***P < 0.001 vs. ACSF; n = 6 in each group.

**Figure 3:**
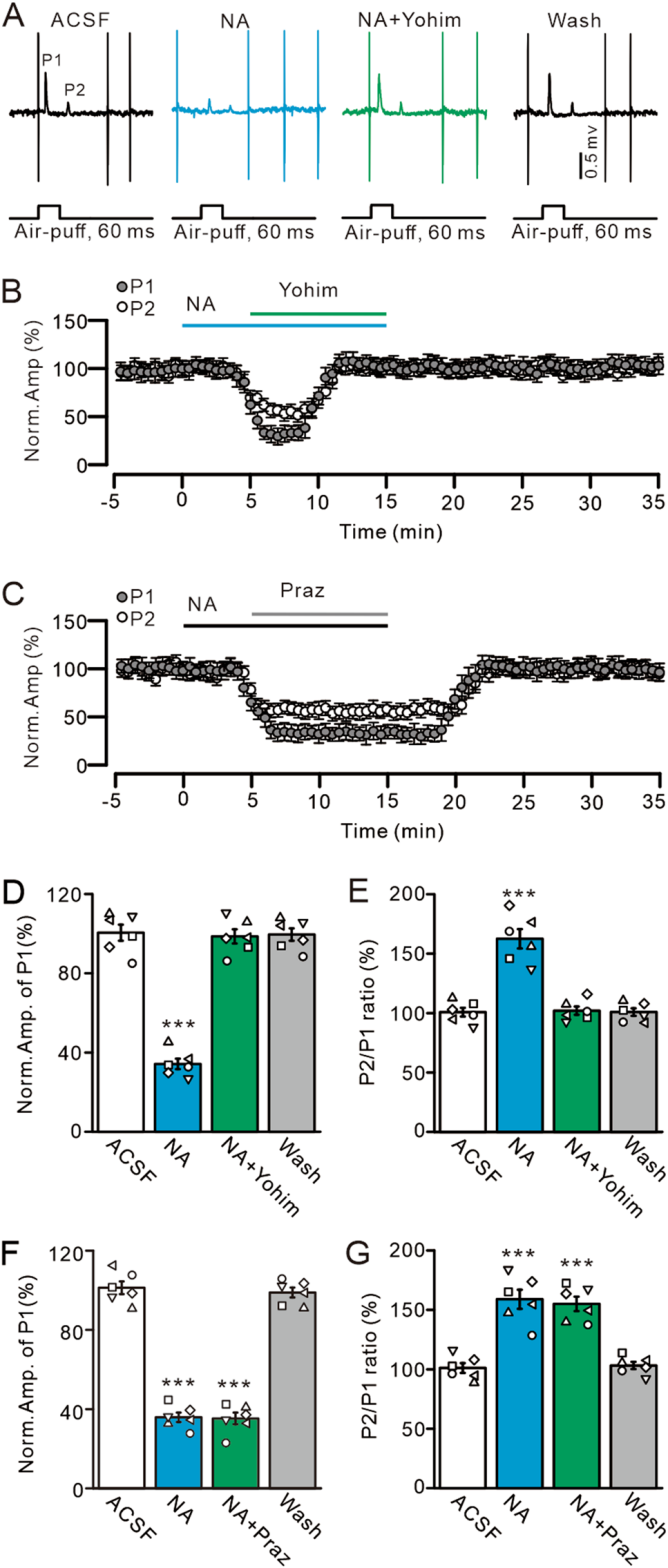
NA depresses MLI-PC synaptic transmission through α2-AR. (**A**) Representative traces showing air-puff stimulation-evoked responses in a cerebellar PC in ACSF, NA (15 µM), NA + Yohimbine (Yohim; 100 µM), and washout. (**B**) Summary of data shows the time course of normalized amplitude of P1 and P2 during treatments with ACSF, NA, NA + Yohim and washout. (**C**) Summary of data shows the time course of normalized amplitude of P1 and P2 during treatments with ACSF, NA (15 µM), NA + Prazosin (Praz; 50 µM) and washout. (**D**,**E**) Bar graphs show the normalized amplitude of P1 (**D**) and the normalized P2/P1 ratio (**E**) in treatments with ACSF, NA, NA + Yohim and washout. (**F**,**G**) Bar graphs show the normalized amplitude of P1 (**F**) and the normalized P2/P1 ratio (**G**) during each treatment, ACSF, NA, NA + Prazosin and washout. ***P < 0.001 vs. ACSF; n = 6 in each group.

**Figure 4:**
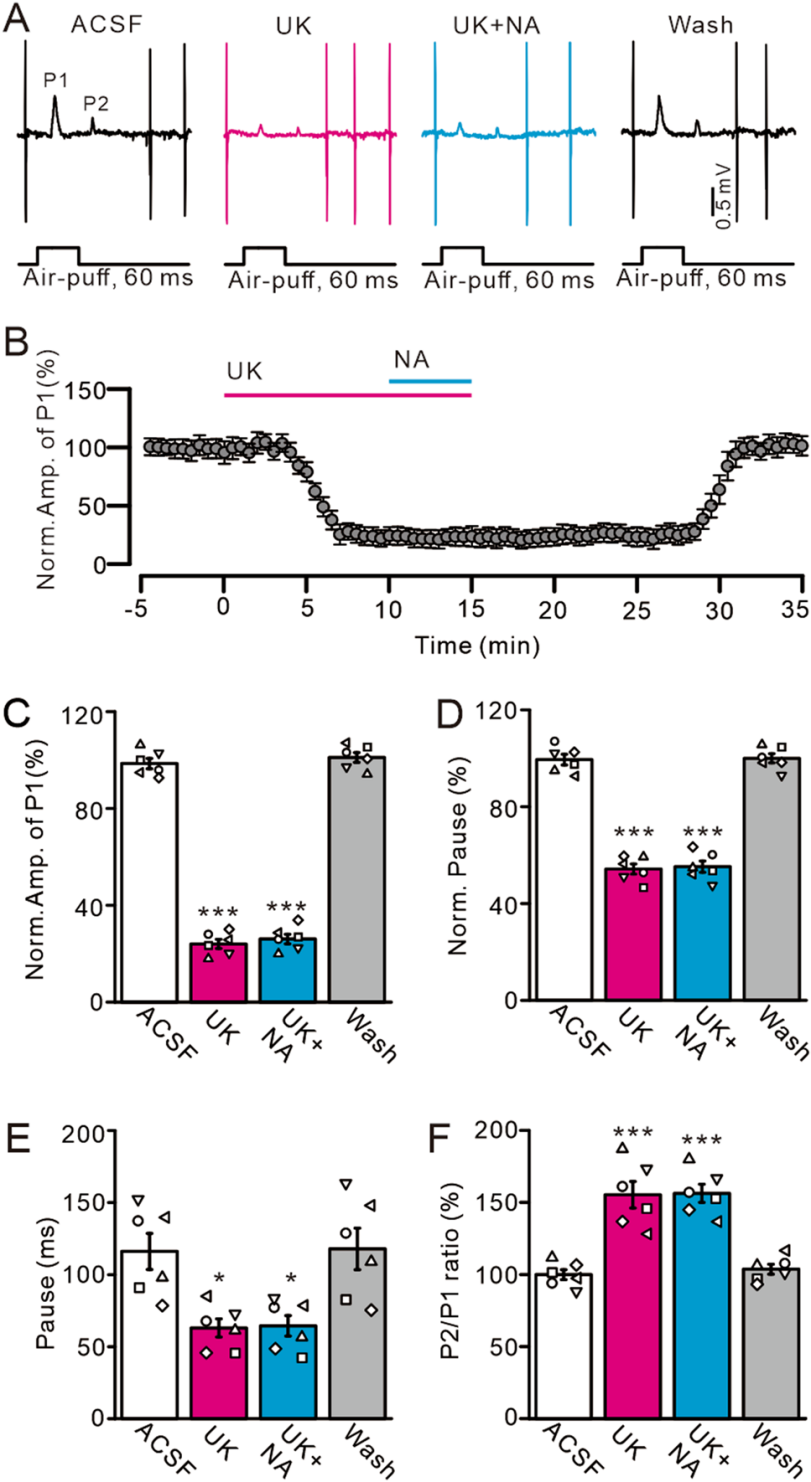
Activation of α2-AR by UK14304 (UK) mimics the NA-induced inhibition of the MLI-PC synaptic transmission. (**A**) Representative cell-attached recording traces showing air-puff stimulation (60 ms; 60 psi)-evoked responses in a cerebellar PC in ACSF, UK 14304 (UK; 1 µM), UK + NA (15 µM) and washout. (**B**) Summary of data shows the time of course of normalized amplitude of P1 during treatments with ACSF, UK, UK + NA and washout. (**C**) Bar graphs show the normalized amplitude of P1 during each treatment. (**D**,**E**) Bar graphs show the normalized pause of SS (**D**) and time of pause (**E**) during each treatment. (**F**) Summary of data showing the normalized P2/P1 ratio in ACSF, UK, UK + NA, and washout. ***P < 0.001 vs. ACSF; *P < 0.05 vs. ACSF; n = 6 in each group.

**Figure 5:**
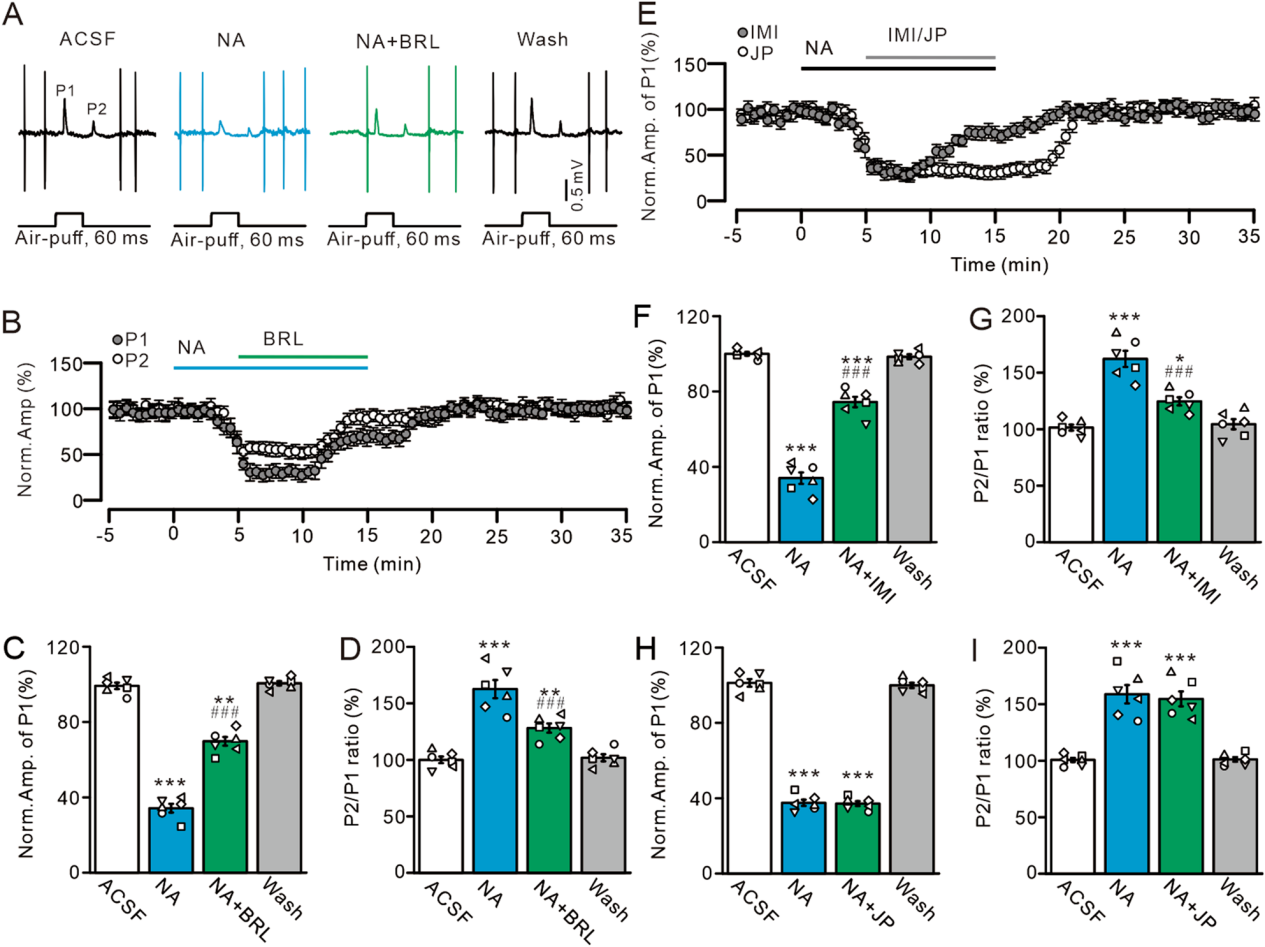
NA inhibits MLI-PC synaptic transmission through α2A- and α2B-AR subtypes. (**A**) Representative cell-attached recording traces showing air-puff stimulation (60 ms; 60 psi)-evoked responses in a cerebellar PC in ACSF, NA (15 µM), NA + BRL44408 (BRL; 50 µM), and washout. (**B**) Summary of data shows the time course of normalized amplitude of P1 and P2 during treatments with ACSF, NA, NA + BRL and washout. (**C**,**D**) Summary of data show the normalized amplitude of P1 (**C**) and the normalized P2/P1 ratio (**D**) during treatments with ACSF, NA, NA + BRL and recovery (washout). (**E**) Summary of data shows the time course of normalized amplitude of P1 during treatments with ACSF, NA (15 µM), NA + Imiloxan (IMI; 50 µM; grey) or NA + JP1302 (JP; 50 µM; white) and washout. (**F**,**G**) Bar graphs show the normalized amplitude of P1 (**F**) and P2/P1 ratio (**G**) in treatments with ACSF, NA, NA + Imiloxan and washout. (**H**,**I**) Bar graphs show the normalized amplitude of P1 (**H**) and P2/P1 ratio (**I**) during treatments with ACSF, NA, NA + JP1302 and washout. *P < 0.05, **P < 0.01 and ***P < 0.001 vs. ACSF; ^#^*P* < 0.05, ^##^*P* < 0.01 and^###^*P* < 0.001 vs. NA; n = 6 in each group.

**Figure 6:**
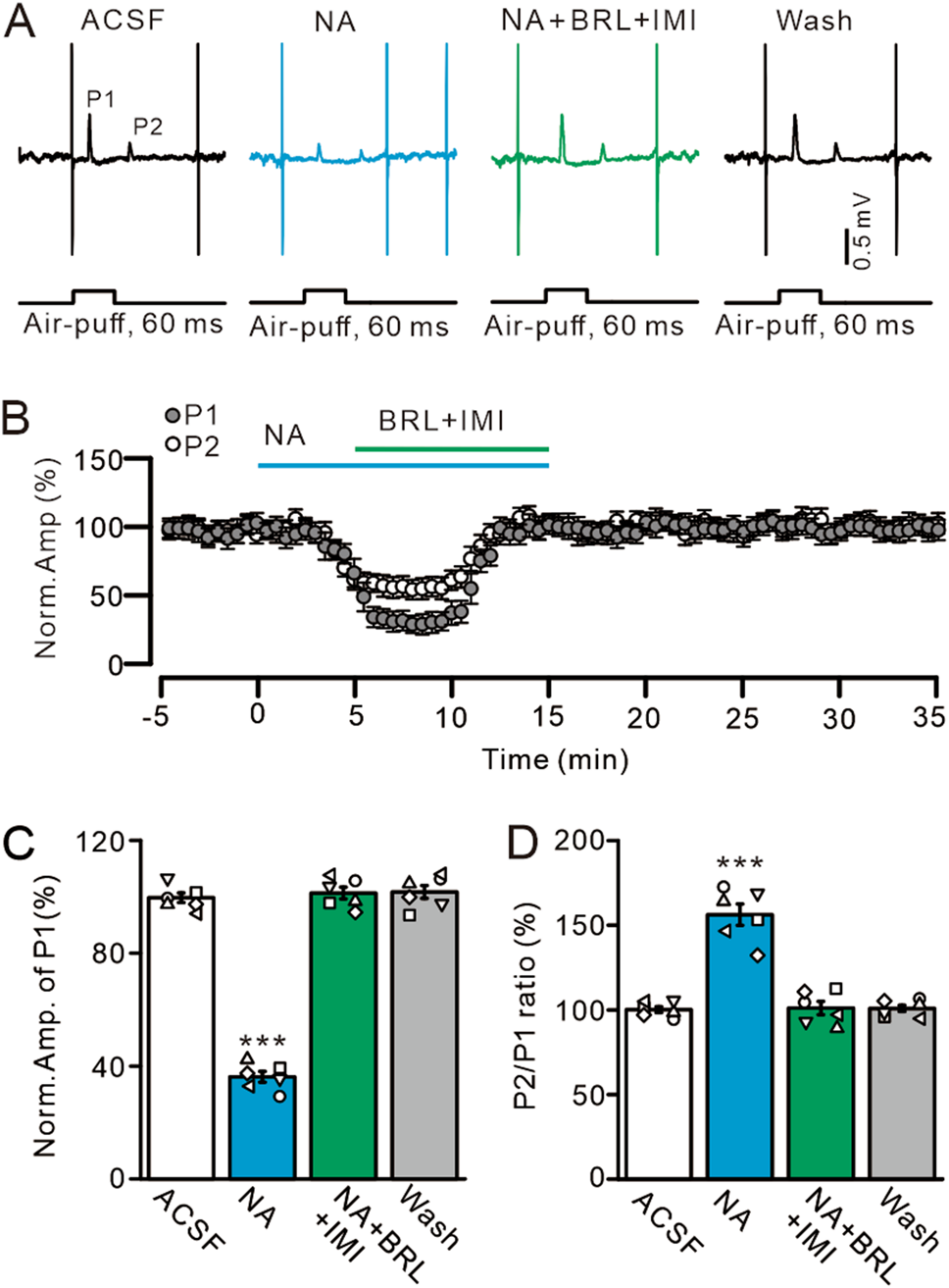
Co-blockade of α2A- and α2B-ARs abolishes NA-induced inhibition of the MLI-PC synaptic transmission. (**A**) Representative cell-attached recording traces showing air-puff stimulation (60 ms; 60 psi)-evoked responses in a cerebellar PC in ACSF, NA (15 µM), NA + BRL (50 µM) + IMI (50 µM), and washout. (**B**) Summary of data showing the time course of normalized amplitude of P1 and P2 in treatments with ACSF, NA, NA + BRL + IMI and washout. (**C**,**D**) Bar graphs show the normalized amplitude of P1 (**C**) and P2/P1 ratio (**D**) in treatments with ACSF, NA, NA + BRL + IMI and washout. ***P < 0.001 vs. ACSF; n = 6 in each group.

**Figure 7:**
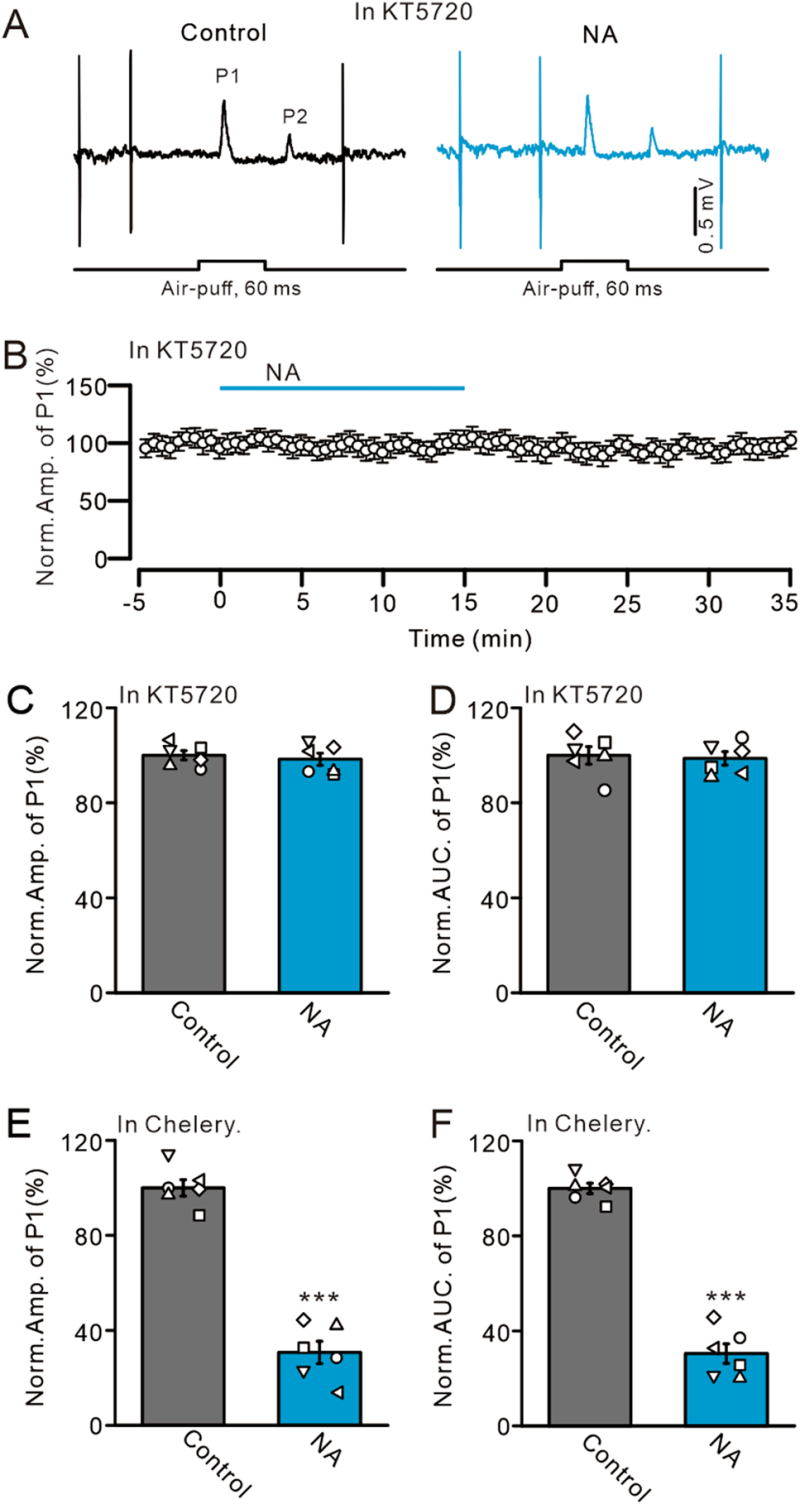
NA inhibits MLI-PC synaptic transmission through a PKA signaling pathway. (**A**) Representative traces showing air-puff stimulation (60 ms; 60 psi -evoked responses in a cerebellar PC in treatments with KT5720 (1 µM), and KT5720 + NA (15 µM). (**B**) Summary of data showing the time course of normalized amplitude of P1 in treatments with KT5720, and KT5720 + NA. (**C**,**D**) Bar graphs show the normalized amplitude (**C**) and AUC (**D**) of P1 during treatments with KT5720, and KT5720 + NA. (**E**,**F**) Bar graphs show the normalized amplitude (**E**) and AUC (**F**) of P1 during treatments with chelerythrine (Chelery, 30 µM), and chelerythrine + NA (15 µM). ***P < 0.001 vs. control; n = 6 in each group.

**Figure 8:**
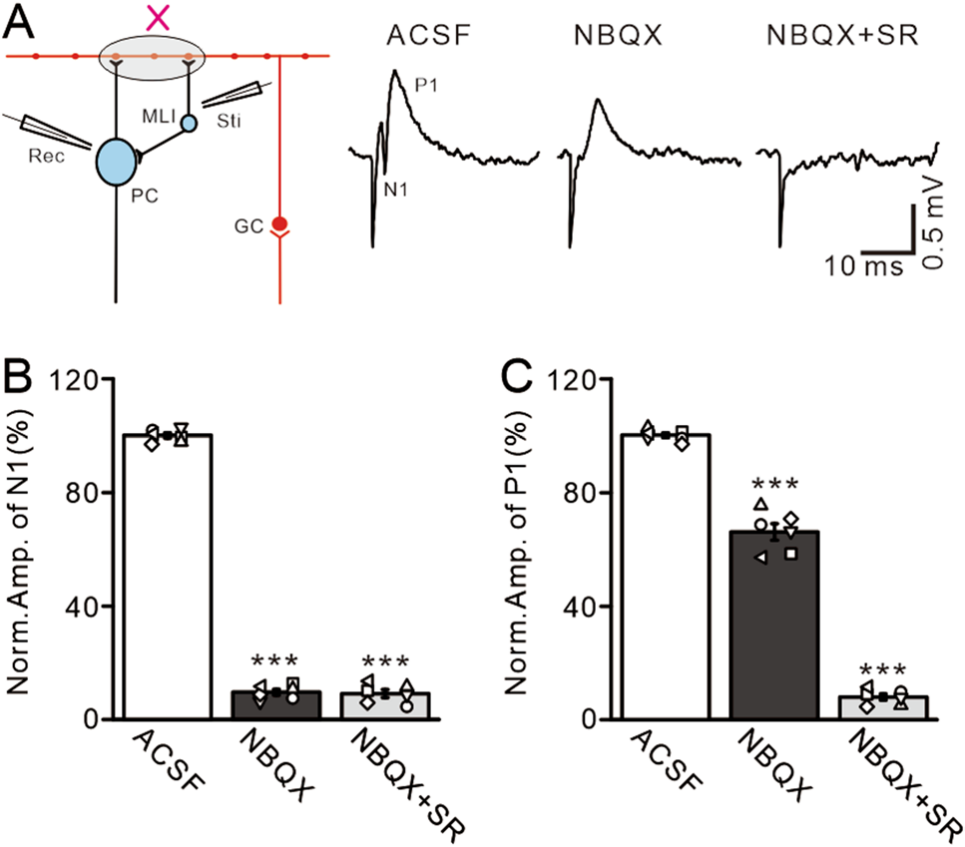
Pharmacological properties of molecular layer electrical stimulation-induced responses of PCs. (**A**) Left: a schematic diagram showing the due electrophysiological recording protocol for the electrical stimulation-evoked MLI-PC synaptic transmission. *Sti* stimulation electrode, *Rec* recording electrode. Right: representative recording traces showing electric stimulation (200 µs, 50–100 μA)-evoked responses in a cerebellar PC in treatments with ACSF, NBQX (50 µM), and NBQX + SR (20 µM). (**B**) Bar graphs show the normalized amplitude of N1 during treatments with ACSF, NBQX, and NBQX + SR. (**C**) Summary of data showing the normalized amplitude of P1 during treatments with ACSF, NBQX, and NBQX + SR. ***P < 0.001 versus ACSF; n = 6 in each group.

**Figure 9:**
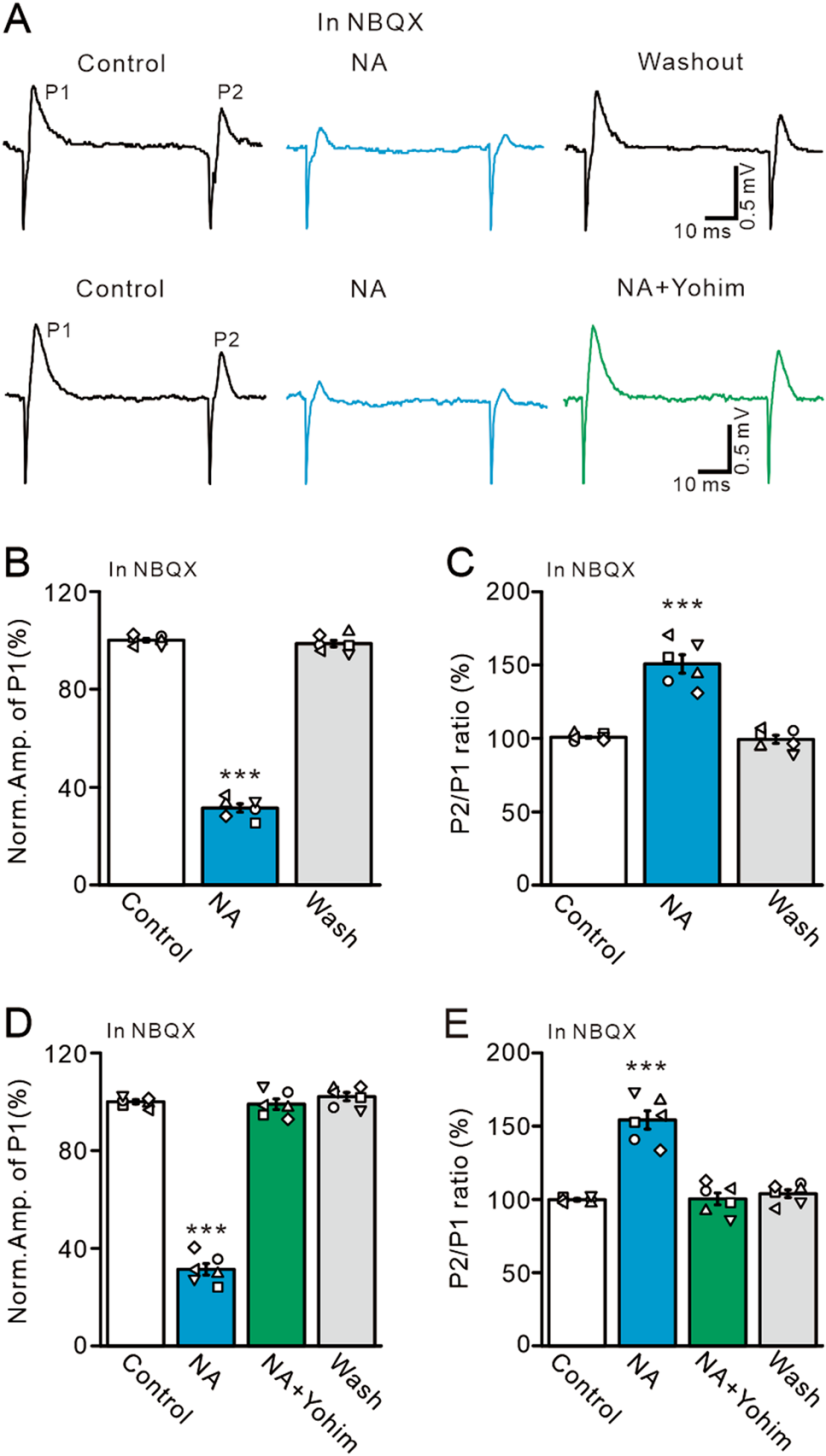
NA inhibits the electrical stimulation-evoked MLI-PC GABAergic synaptic responses. (**A**) In the presence of NBQX (50 µM); upper: representative traces showing the electrical stimulation (200 µs, 50–100 μA)-evoked responses in a cerebellar PC under conditions of control, NA (15 µM), and washout. Lower: representative traces showing electric stimulation-evoked responses in a cerebellar PC under conditions of control, NA (15 µM), and NA + Yohim (100 μM). (**B**,**C**) Bar graphs show the normalized amplitude of P1 (**B**) and P2/P1 ratio (**C**) under conditions of control, NA, and washout. (**D**,**E**) Bar graphs show the normalized amplitude of P1 (**D**) and P2/P1 ratio (**E**) under conditions of control, NA, NA + Yohim and washout. ***P < 0.001 versus control; n = 6 in each group.

Electrophysiological data were analyzed using Clampfit 10.4 software (Molecular Devices, Foster City, CA). Paired-pulse ratio was calculated as amplitude of P2 divided by amplitude of P1. Data were normalized to baseline and used for further analyses. All values were expressed as the mean ± SEM, and differences were evaluated by the Student’s paired t-test (Figs. 1, 7) and One-way ANOVA (Figs. 2, 3, 4, 5, 6, 8, 9) using SPSS software (Chicago, IL). *P* values below 0.05 were considered statistically significant.

### Ethical approval

The experimental procedures were approved by the Animal Care and Use Committee of the Yanbian University. Permission No. is SYXK (Ji) 2011-006. All the experimental methods were in accordance with the animal welfare guidelines of the U.S. National Institutes of Health, and the Animal Research: Reporting in Vivo Experiments (ARRIVE; https://arriveguidelines.org).

## Results

### Effects of NA on facial stimulation-evoked MLI-PC synaptic transmission

A total of 166 neurons were identified as PCs that exhibited both simple spike (SS) and complex spike firing activity. Under cell-attached recording conditions, air-puff stimulation (60 ms; 60 psi) on the ipsilateral whisker pad induced inhibitory components (P1 and P2), followed by a pause in SS firing (Fig. 1A,C). Consistent with our previous studies^5–7^, P1 and P2 were identified as forms of facial stimulation-evoked MLI-PC GABAergic synaptic transmission. ML microapplication of NA (15 µM) significantly inhibited P1 and P2 (Fig. 1A,B). In addition, NA decreased the spontaneous SS firing rate of cerebellar PCs (Fig. 1C). In the presence of NA, the normalized amplitude of P1 was 31.66 ± 2.17% that of baseline (artificial cerebrospinal fluid [ACSF]: 100.0 ± 3.03%; *P* < 0.0001; n = 6; Fig. 1D), the normalized duration of the pause in SS was 56.72 ± 2.53% that of baseline (100.0 ± 2.13%; *P* < 0.0001; n = 6; Fig. 1E), and the actual pause duration was 64.29 ± 8.45 ms (ACSF: 114.9 ± 15.59 ms; *P* = 0.0012; n = 6; Fig. 1F). PCs exhibited regular SS firing, with a mean firing rate of 16.37 ± 2.05 Hz (ACSF: 31.41 ± 2.63 Hz; *P* < 0.0001; n = 6). However, the normalized P2/P1 ratio was 159.15 ± 7.76% that of baseline during the application of NA (ACSF: 99.5 ± 3.1%; *P* < 0.001; n = 6; Fig. 1G). NA reduced the amplitude of P1 in a concentration-dependent manner. The half-inhibitory concentration of NA was 10.94 µM (Fig. 1H). In addition, there was no significant difference in the amplitude of facial stimulation-evoked P1 between females and males in the presence of NA (P = 0.39; n = 3 mice in the female and male groups (data not shown). Taken together, the results indicate that ML microapplication of NA induces a concentration-dependent decrease in facial stimulation-evoked MLI-PC synaptic transmission.

### NA-induced depression of MLI-PC synaptic transmission was mediated by α-ARs

α- and β-ARs, which modulate sensory information processing and motor coordination, are widely expressed in the cerebellar cortex^18^, and therefore we examined whether NA-induced inhibition of facial stimulation-evoked MLI-PC synaptic transmission occurred through α-ARs. As shown in Fig. 2, NA (15 µM) inhibited MLI-PC synaptic transmission; this effect was abolished by a non-selective α-AR antagonist (phentolamine, 100 µM) (Fig. 2A,B). In the presence of NA and phentolamine, the normalized amplitude of P1 increased from 35.13 ± 2.09% (NA alone) to 101.52 ± 2.81% that of baseline (*P* < 0.0001 vs. NA alone; n = 6; Fig. 2D), and the normalized P2/P1 ratio decreased from 155.13 ± 8.37% (NA alone) to 100.85 ± 3.66% that of baseline (*P* < 0.0001 vs. NA alone; n = 6; Fig. 2E). The mean frequency of SS firing increased from 15.74 ± 1.83 Hz (NA alone) to 29.88 ± 2.65 Hz (ACSF: 29.75 ± 3.26 Hz; *P* = 0.0013 vs. NA alone; n = 6; not shown). Furthermore, we used a non-selective β-AR blocker (propranolol, 100 µM) to determine whether NA inhibited the MLI-PC synaptic response through the activation of β-ARs. The application of propranolol failed to abolish NA-induced inhibition of MLI-PC synaptic transmission (Fig. 2C). In the presence of NA and propranolol, the normalized amplitude of P1 was 30.52 ± 2.59% (n = 6) that of baseline, which was similar to that of NA alone (31.85 ± 3.42%; *P* = 0.95; n = 6; Fig. 2F), and the P2/P1 ratio was 156.76 ± 9.85% that of baseline, which was also similar to that of NA alone (157.89 ± 7.16%; *P* = 0.92; n = 6; Fig. 2G). Moreover, the mean frequency of SS firing was 15.53 ± 1.85 Hz, and there was no significant change compared with NA alone (15.88 ± 2.09 Hz; *P* = 0.9; n = 6; not shown).

### NA inhibited facial stimulation-evoked MLI-PC synaptic transmission through activation of α2-ARs

NA depresses climbing fiber-PC and MF-GC synaptic transmission via α2-ARs^26–28^, and therefore we applied the α2-AR antagonist yohimbine (100 µM) to determine whether NA can inhibit MLI-PC synaptic transmission through activation of α2-ARs. The results showed that application of yohimbine completely abolished the inhibitory effect of NA on P1 (Fig. 3A,B). In the presence of NA and yohimbine, the normalized amplitude of P1 increased from 34.23 ± 2.62% (NA, 15 µM) to 98.62 ± 3.55% that of baseline (*P* < 0.0001 vs. NA alone; n = 6; Fig. 3D), and the P2/P1 ratio decreased from 162.43 ± 8.13% (NA, 15 µM) to 101.99 ± 3.45% that of baseline (*P* < 0.001 vs. NA alone; n = 6; Fig. 3E). Moreover, the mean frequency of SS firing increased from 14.2 ± 1.79 Hz (NA alone) to 26.31 ± 1.66 Hz (ACSF: 26.19 ± 2.65 Hz; *P* < 0.0001 vs. NA alone; n = 6; not shown). These results suggest that NA suppresses facial stimulation-evoked MLI-PC synaptic transmission through presynaptic α2-ARs. However, application of the α1-AR antagonist prazosin (50 µM) had less effect on the NA-induced inhibition of MLI-PC synaptic transmission (Fig. 3C). In the presence of NA (15 µM) and prazosin (50 µM), the normalized amplitude of P1 was 35.29 ± 2.94% (n = 6) that of baseline (ACSF: 100.0 ± 3.19%; *P* < 0.001; n = 6), which was similar to that of NA alone (35.87 ± 2.38%; *P* = 0.78; n = 6; Fig. 3F). The normalized P2/P1 ratio was 155.02 ± 6.07% (NA + prazosin; n = 6) that of baseline (ACSF: 100.0 ± 4.11%; *P* < 0.001; n = 6), which was not significantly different from that of NA alone (158.91 ± 8.08%; *P* = 0.93; n = 6; Fig. 3G). The mean frequency of SS firing was 16.71 ± 1.61 Hz and there was no significant change compared with NA alone (17.13 ± 1.71 Hz; *P* = 0.85; n = 6; not shown).

We also assessed the effect of a highly selective α2-AR agonist, UK14304, on MLI-PC synaptic transmission. Bath application of UK14304 (1 µM) in ML induced a time-dependent decrease of facial stimulation-induced MLI-PC synaptic transmission (Fig. 4A,B). In the presence of UK14304, the normalized amplitude of P1 was 24.72 ± 2.25% that of baseline (ACSF: 100.0 ± 2.15%; *P* < 0.0001; n = 6; Fig. 4C). In addition, the normalized duration of the pause in SS was 54.69 ± 2.09% that of baseline (ACSF: 100.0 ± 2.21%; *P* < 0.001; n = 6; Fig. 4D), the actual pause duration was 62.9 ± 6.36 ms (ACSF: 116.02 ± 12.5 ms; *P* = 0.01; n = 6; Fig. 4E), and the normalized P2/P1 ratio was 155.37 ± 9.21% that of baseline (ACSF: 100.0 ± 3.47%; *P* < 0.001; n = 6; Fig. 4F). PCs exhibited regular SS firing at a mean rate of 14.46 ± 1.41 Hz in the presence of UK14304, which was significantly lower than that in ACSF (ACSF: 30.52 ± 2.57 Hz; *P* < 0.0001; n = 6). Notably, bath application of UK14304 prevented the effect of microinjection of NA on facial stimulation-induced MLI-PC synaptic transmission (Fig. 4A,B). In the presence of UK14304 and NA, the normalized amplitude of P1 was 25.81 ± 1.64% that of baseline, which was similar to that of UK alone (*P* = 0.96; Fig. 4C). Additionally, the normalized duration of the pause for SS was 55.65 ± 2.6% that of baseline, which was similar to that of UK alone (*P* = 0.89; Fig. 4D), and the actual pause duration was 64.37 ± 7.15 ms, which was not significantly different from that of UK alone (*P* = 0.99; Fig. 4E). Finally, the normalized P2/P1 ratio was 156.31 ± 6.31% that of baseline, which was not significantly different than that of UK alone (*P* = 0.88; Fig. 4F). These findings indicate that pharmacological activation of α2-ARs depresses facial stimulation-evoked MLI-PC synaptic transmission and prevents NA from further depressing synaptic transmission.

### NA depressed facial stimulation-evoked MLI-PC synaptic transmission via α2-AR subtypes

The α2-AR family includes α2A-, α2B- and α2C-AR subtypes, which are expressed in the cerebellum^29–35^. The application of an α2A-AR antagonist, BRL44408 (BRL, 50 µM), partly abolished the NA-induced depression of MLI-PC synaptic transmission (Fig. 5A,B). In the presence of NA and BRL, the normalized amplitude of P1 increased from 34.24 ± 2.29% (NA, 15 µM) to 69.77 ± 2.31% that of baseline (*P* < 0.001 vs. NA alone; *P* = 0.0073 vs. ACSF; n = 6; Fig. 5C), and the normalized P2/P1 ratio decreased from 162.56 ± 8.05% (NA, 15 µM) to 128.13 ± 4.09% that of baseline (ACSF: 100 ± 2.91%; *P* < 0.001 vs. NA alone; *P* = 0.0038 vs. ACSF; n = 6; Fig. 5D). The mean frequency of SS firing increased from 15.01 ± 1.39 Hz (NA alone) to 23.01 ± 2.34 Hz (ACSF: 28.09 ± 2.31 Hz; *P* = 0.0015 vs. NA alone; *P* < 0.001 vs. ACSF; n = 6; not shown). In the presence of NA and an α2B-AR antagonist (imiloxan, 50 µM) (Fig. 5E), the normalized amplitude of P1 increased from 34.02 ± 3.05% (NA, 15 µM) to 74.41 ± 2.78% that of baseline (ACSF: 100 ± 0.95%; *P* < 0.001 vs. NA alone; *P* < 0.001 vs. ACSF; n = 6; Fig. 5F). Moreover, the normalized P2/P1 ratio was reduced from 163.23 ± 7.12% (NA, 15 µM) to 124.58 ± 3.58% that of baseline (ACSF: 101.39 ± 2.66%; *P* < 0.001 vs. NA alone; *P* = 0.013 vs. ACSF; n = 6; Fig. 5G). Moreover, the mean frequency of SS firing increased from 14.81 ± 2.29 Hz (NA alone) to 21.99 ± 2.49 Hz (ACSF: 26.39 ± 3.32 Hz; *P* = 0.0027 vs. NA alone; *P* < 0.001 vs. ACSF; n = 6; not shown). In the presence of NA and an α2C-AR antagonist (JP1302, 50 µM) (Fig. 5E), the normalized amplitude of P1 was 37.11 ± 1.31% (NA + JP1302; n = 6) that of baseline, which was not significantly different from that of NA alone (37.58 ± 1.69%; P = 0.9; n = 6; Fig. 5H). The normalized P2/P1 ratio was 154.68 ± 6.59% that of baseline, which was similar to that of NA alone (159.16 ± 8.09%; *P* = 0.96; n = 6; Fig. 5I). Moreover, the mean frequency of SS firing was 17.38 ± 2.09 Hz, which was not significantly different than that of NA alone (17.01 ± 1.22 Hz; *P* = 0.97; n = 6 [data not shown]).

We then used a mixture of BRL (50 µM) and imiloxan (50 µM) to examine whether microapplication of NA depressed MLI-PC synaptic transmission through both α2A- and α2B-ARs. In the presence of a mixture of BRL (50 µM) and imiloxan (50 µM), microapplication of NA (15 µM) failed to depress MLI-PC synaptic transmission (Fig. 6A,B). In the presence of BRL, imiloxan, and NA, the normalized amplitude of P1 increased from 36.16 ± 1.91% (NA, 15 µM) to 101.34 ± 2.09% that of baseline (ACSF: 100.0 ± 1.96%; *P* < 0.001 vs NA alone; *P* = 0.76 vs. ACSF; n = 6; Fig. 6C). Additionally, the normalized P2/P1 ratio decreased from 156.05 ± 6.23% (NA, 15 µM) to 100.94 ± 3.93% that of baseline (ACSF: 100.0 ± 0.69%; *P* < 0.001 vs. NA alone; *P* = 0.69 vs. ACSF; n = 6; Fig. 6D). The mean frequency of SS firing was increased from 16.6 ± 2.12 Hz (NA alone) to 32.41 ± 2.34 Hz, which was similar to that in ACSF (ACSF: 33.29 ± 3.15 Hz; *P* < 0.0001 vs. NA alone; *P* = 0.94 vs. ACSF; n = 6; not shown). These findings suggested that NA-induced inhibition of facial stimulation-evoked MLI-PC synaptic transmission is mediated by both α2A- and α2B-ARs in vivo in mice.

### NA inhibited facial stimulation-evoked MLI-PC synaptic transmission via the PKA signaling pathway

Previous studies demonstrated that activation of α2-ARs inhibits adenylate cyclase activity, reduces cyclic adenosine monophosphate (cAMP), and exerts physiological effects through the PKA signaling pathway^19, 36^. In this study, when PKA was inhibited by KT5720 (1 µM), the application of NA (15 µM) failed to inhibit facial stimulation-evoked MLI-PC synaptic transmission (Fig. 7A,B). In the absence of PKA activity, the normalized amplitude of P1 was 98.35 ± 2.54% that of baseline (control: 100.0 ± 1.96%; *P* = 0.61; n = 6; Fig. 7C), and the normalized area under the curve of P1 was 98.72 ± 2.86% that of baseline (control: 100.0 ± 3.69%; *P* = 0.79; n = 6; Fig. 7D). There was no significant change in the mean frequency of SS firing compared with control (not shown). In contrast, inhibition of PKC by chelerythrine (30 µM) failed to abolish the NA (15 µM)-induced inhibition of MLI-PC synaptic transmission. In the absence of PKC activity, the normalized amplitude of P1 was 30.74 ± 4.71% that of baseline (control: 100.0 ± 3.46%; *P* < 0.001; n = 6; Fig. 7E), which was similar to that of NA alone (31.66 ± 2.17%; *P* = 0.79). The normalized area under the curve of P1 was 30.43 ± 4.08% that of baseline (control: 100.0 ± 2.21%; *P* < 0.001; n = 6; Fig. 7F), which was not significantly different from that of NA alone (30.76 ± 1.46%; *P* = 0.78). In addition, the mean frequency of SS firing was not significant different than that of NA alone (not shown). These results indicate that inhibiting PKA abolishes the NA-induced decrease in MLI-PC synaptic transmission, in turn suggesting that NA inhibits facial stimulation-evoked MLI-PC synaptic transmission via the PKA signaling pathway.

### NA inhibited electrical stimulation of ML-evoked MLI-PC GABAergic synaptic transmission

The facial stimulation-evoked MLI-PC synaptic transmission occurred via the MF-GC-parallel fiber pathway, and therefore cerebellar ML application of NA inhibited the MLI-PC synaptic response via direct inhibition of MLI-PC synaptic transmission, or indirectly by depressing parallel fiber-MLI synaptic transmission. Our results showed that NA depressed facia stimulation-evoked MLI-PC GABAergic synaptic transmission, accompanied by an increase in the P2/P1 ratio, suggesting that NA directly inhibited MLI-PC synaptic transmission by reducing GABA release at MLI-PC synapses. We further isolated the ML stimulation-evoked MLI-PC GABAergic synaptic response by bath application of an AMPA receptor antagonist, NBQX (50 µM). Electrical stimulation of the ML (200 µs, 50–100 µA) evoked a response in cerebellar PCs, which expressed a negative component (N1) followed by a positive component (P1) (Fig. 8A). Cerebellar surface perfusion of NBQX completely blocked N1, indicating that N1 is an AMPA receptor-mediated glutamatergic component (Fig. 8B). Because AMPA receptor blockade abolished parallel fiber-PC synaptic transmission, we did not employ an NMDA receptor antagonist in this experiment. Notably, P1 was abolished by application of a GABA_A_ receptor antagonist, gabazine (20 µM), indicating that P1 arises from an MLI-PC GABAergic response (Fig. 8C). The electrical stimulation-evoked MLI-PC response was inhibited by microapplication of 15 µM NA, which was reversed by bath application of an α2-AR antagonist, yohimbine (100 µM; Fig. 9A). In the presence of NBQX and NA, the normalized amplitude of P1 was 31.51 ± 1.71% that of baseline (*P* < 0.0001; n = 6; Fig. 9B), and the normalized P2/P1 ratio was 150.66 ± 6.28% that of baseline (*P* < 0.001; n = 6; Fig. 9C). In the presence of NBQX, NA and yohimbine, the normalized amplitude of P1 increased from 31.36 ± 2.35% (NA, 15 µM) to 99.02 ± 2.16% that of baseline (*P* < 0.0001 vs. NA alone; n = 6; Fig. 9D), and the normalized P2/P1 ratio decreased from 154.24 ± 6.25% (NA, 15 µM) to 100.35 ± 4.07% that of baseline (*P* < 0.0001; n = 6; Fig. 9E). These results indicate that NA directly inhibits MLI-PC synaptic transmission via α2-ARs, suggesting that NA inhibits facial stimulation-evoked MLI-PC synaptic transmission by reducing GABA release at MLI-PC synapses.

## Discussion

In this study, cerebellar ML microapplication of NA elicited concentration-dependent depression of facial stimulation-evoked MLI-PC GABAergic synaptic transmission, accompanied by an increase in the P2/P1 ratio, through activation of α2-ARs. The NA-induced suppression of MLI-PC synaptic transmission was inhibited by blockade of α2A- or α2B-ARs, but not by antagonism of α2C-ARs, and it was completely blocked by a mixture of α2A- and 2B-AR antagonists. NA-induced depression of MLI-PC synaptic transmission was abolished by inhibiting PKA, but not by inhibiting PKC. Moreover, electrical stimulation evoked MLI-PC GABAergic synaptic transmission in the absence of AMPA receptor activity, which was depressed by NA via α2-ARs. These results indicate that NA inhibits facial stimuli-evoked MLI-PC synaptic transmission through the presynaptic α2-AR/PKA pathway, and that NA plays a critical modulatory role in MLI-PC synaptic transmission in vivo in mice.

### NA depresses facial stimulation-induced MLI-PC synaptic transmission via α2-ARs

Sensory information is transferred to the cerebellar cortex through the MF-GC-parallel fiber and climbing fiber pathways, and is then integrated by PCs to generate motor outputs^2–4^. Anatomical studies indicate that NAergic fibers originate from the LC and project to all layers of the cerebellar cortex^13, 37^. Importantly, NAergic fibers form varicosities near the dendrites of PCs^38^, which indicates that NAergic afferents are critical for the regulation of PC activity and synaptic transmission in the cerebellar cortex^9, 11^. Electrophysiological studies in living animals have demonstrated that NA depressed neuronal activity in the cerebellar cortex via the activation of α2-ARs^26, 27^ and also inhibited facial stimulation-evoked MF-GC synaptic transmission^28^. The present results showed that cerebellar surface micro-application of NA inhibits either facial stimulation- or electric stimulation-evoked MLI-PC synaptic transmission in a concentration-dependent manner, accompanied by a significant increase in the P2/P1 ratio. These results suggest that NA inhibits MLI-PC synaptic transmission by reducing GABA release via the activation of ARs.

Previous studies have shown that NA differentially modulates neurotransmitter release and synaptic transmission in the cerebellar cortex by activating various subtypes of ARs at pre- or postsynaptic sites^29^. NA reduces the excitability of MLIs through presynaptic α2-ARs^20, 21^, and activation of α2-ARs inhibits GABA release in the cerebellar cortex through either pre-or postsynaptic sites^45^. The activation of presynaptic α2-ARs reduces GABA release from histaminergic neurons of the tuberomammillary nucleus in vitro in rats^47^. We previously demonstrated that NA depresses facial stimulation-evoked MF-GC synaptic transmission and climbing fiber-PC synaptic transmission via α2-ARs in vivo in mice, indicating that α2-ARs play important modulatory roles in synaptic transmission in the cerebellar cortex^26–28^. Consistent with previous studies^26–28, 46, 47^, the present results showed that an α2-AR antagonist, but not α1-AR or β-AR antagonists, abolished NA-induced inhibition of facial stimulation-evoked MLI-PC synaptic transmission, suggesting that NA depresses MLI-PC synaptic transmission through α2-ARs. Notably, a highly selective α2-AR agonist, UK14304, mimicked the NA-induced suppression of MLI-PC synaptic transmission and overwhelmed the effect of NA on synaptic responses; these results indicate that NA reduced MLI-PC synaptic transmission through α2-ARs.

Previous studies demonstrated that NA facilitated GABAA receptor function through a postsynaptic β-AR mediated, cAMP-dependent cascade in cerebellar PCs^42, 43^ and enhanced GABAergic synaptic transmission by activating presynaptic β2-AR in cerebellar MLI-PC synapses in cerebellar slices^41, 44^. Through α1-ARs expressed in the presynaptic terminals and soma, NA also enhances inhibitory neurotransmitter release in dendritic domains of cerebellar PCs in vitro in rodents^20, 46^. However, this study showed that NA reduced facial stimulation-evoked MLI-PC synaptic transmission through α2-ARs. The present results are contrary to those of previous studies, which may be attributable to the differences between in vivo and in vitro experimental conditions. The cerebellar cortex receives noradrenergic inputs from the LC, which may release a certain amount of NA during experimental processes^9, 10, 39^. The NA-induced depression of facial stimulation-evoked MLI-PC synaptic transmission observed herein was not dependent on beta- or α1-ARs, possibly because of the status of these AR subtypes during the experimental process (e.g., AR activation saturation or desensitization). Endogenous NA might activate and desensitize beta- and α1-ARs of the MLI membrane and axonal terminals such that exogenous NA cannot activate them. Thus, the additional application of NA inhibits facial stimulation-evoked MLI-PC synaptic transmission by activating α2-AR. However, further experiments are needed to understand the mechanisms of NA-induced inhibition of facial stimulation-evoked MLI-PC synaptic transmission in vivo in mice.

Although the α2A-AR is a major presynaptic receptor subtype regulating neurotransmitter release, α2B- and α2C-ARs also function as presynaptic autoreceptors inhibiting neurotransmitter release^48–50^. In the cerebellum, α2A- and α2B-AR mRNA is expressed in PCs, GCs, and MLIs^31, 32, 35, 51^. The α2C-AR mRNA is transiently expressed in the granular layer and ML during GC development^52^. Immunohistochemical studies showed that α2A-AR was abundantly expressed in PCs and GCs^33, 34^. Moreover, α2C-AR immunoreaction products are scattered throughout the granular layer and ML of the rat cerebellum^53^. Knockout of the α2A-AR gene impaired motor coordination in mice^54^. In histaminergic neurons of the tuberomammillary nucleus, NA-induced inhibition of GABAergic synaptic transmission was significantly blocked by a selective α2A-AR antagonist, but not by a selective α2B or α2C adrenoceptor antagonist, indicating that α2A-AR mediates NA-induced inhibition of GABAergic synaptic transmission^55^. However, the present results showed that NA-induced inhibition of MLI-PC synaptic transmission was completely abolished by a mixture of α2A- and α2B-AR antagonists, indicating that both α2A- and α2B-AR are involved in the NA-induced inhibition of MLI-PC synaptic transmission. In the cerebellar cortex, basket-type MLIs are usually found in the inner third of the ML, and their stomata are close by or within the PC layer. They are characterized by basket-like structures with axonal arborizations that envelop PC somas, forming pincer synapses and providing strong basket-type somatic inhibition of PCs^7, 56–58^. Moreover, immunohistochemical results showed that both α2A- and α2B-ARs are abundantly expressed by MLIs, including on somas and axon terminals^29–35^. Our electrophysiological results showed NA-induced inhibition of GABAergic synaptic transmission accompanied by an increase in the P2/P1 ratio through activation of α2A- and α2B-ARs. There are two plausible mechanisms underlying NA-induced inhibition of MLI-PC synaptic transmission through α2A- and α2B-ARs. First, NA can inhibit the neuronal firing activity of MLIs by activating α2A- and α2B-ARs on the soma, leading to a decrease in GABA release at their terminals and inhibition of MLI-PC synaptic transmission. Second, NA can also downregulate the release of GABA by directly activating α2A- and α2B-ARs at MLI axonal terminals, leading to a decrease in GABA release and MLI-PC synaptic transmission.

### NA-induced inhibition of MLI-PC synaptic transmission via the PKA signaling pathway

The α2-AR is a Gi/o-coupled metabolic receptor^36, 59–61^ that negatively regulates the activity of adenylyl cyclases and inhibits voltage-gated Ca^2+^ channel activity^60^. Activation of presynaptic α2-ARs inhibits N-type calcium channels, neurotransmitter release, and PKA activity, which in turn decreases phosphorylation of receptors^61^. Activation of α2-ARs suppresses the production of cAMP-dependent PKA; in turn, this activates protein phosphatase 1, which inhibits synaptic transmission^62, 63^ and reduces presynaptic glutamate release from mitral cells through Gi/o-protein-mediated inhibition of Ca^2+^ channels in the mouse olfactory bulb^64^. In the cerebellar cortex, NA reduces glutamate release at CF-PC synapses via a presynaptic α2-AR-PKA signaling pathway in vitro in mouse^27^. The present results demonstrate that the NA-induced suppression of facial stimulation-evoked MLI-PC synaptic transmission is prevented by inhibition of PKA, suggesting that NA inhibits this form of synaptic transmission via a α2-AR/PKA signaling pathway. In addition, inhibition of PKC fails to prevent the NA-induced suppression of MLI-PC synaptic transmission, suggesting that the inhibition is not dependent on the PKC signaling pathway. We also studied the effect of NA on the facial stimulation-evoked MLI-PC synaptic transmission using urethane anesthetized mice. Although most of anesthesia is known to promote inhibition, urethane produces inhibition of neuronal excitability by activation of the barium-sensitive potassium leak conductance, but did not alter excitatory glutamate-mediated or inhibitory GABAA- or GABAB-mediated synaptic transmission. Neither the amplitude nor decay time constant of GABAA- or GABAB-mediated monosynaptic inhibitory postsynaptic currents were altered by urethane, nor was the frequency of spontaneous inhibitory postsynaptic currents^40^. Therefore, urethane anesthesia should have less effect on facial stimulation-evoked MLI-PC GABAergic synaptic transmission in vivo in mice.

Taken together, the present results indicate that NA activates α2A- and α2B-ARs, resulting in depression of facial stimulation-evoked MLI-PC synaptic transmission through a presynaptic α2-AR/PKA signaling pathway. This suggests that adrenergic neurons in the LC could control sensory information integration in PCs by modulating MLI-PC synaptic transmission in the cerebellar cortex.

### Supplementary Information

Supplementary Information.

## Data Availability

The datasets generated and analyzed during the current study are available from the corresponding author on reasonable request.
