# Supplementary material for: Noradrenaline depresses facial stimulation-evoked cerebellar MLI-PC synaptic transmission via α2-AR/PKA signaling cascade in vivo in mice
Source: Sci Rep. 2023 Sep 23;13:15908. doi: 10.1038/s41598-023-42975-5 (PMC10517918; doi:10.1038/s41598-023-42975-5)
Supplement: Supplementary file 1 — Supplementary Information. [file 41598_2023_42975_MOESM1_ESM.pdf]

Supplementary Material

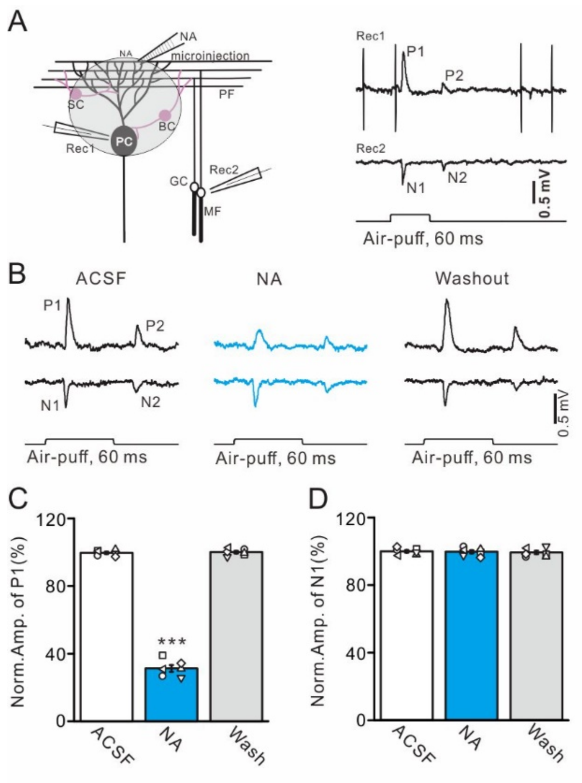

Supplemental Figure 1. Microapplication of NA in molecular layer depresses facial stimulation-evoked MLI-PC, but did less affect the mossy fiber (MF)-granule cell (GC) synaptic transmission. (A) Left: A schematic diagram showing the due electrophysiological recording protocol performed in cerebellar cortical PC layer (PCL) (depth: 200 μm; Rec1) and granular layer (GL) (depth: 300 μm; Rec2). NA was microinjected in the molecular layer by a micropump. Right: Representative cell-attached responses (PC) and field potential responses (GL) simultaneously recorded in a cerebellar PC (Rec1) and GL (Rec2) in response to air-puff (60 ms, 60 psi) stimulation of ipsilateral whisker pad. **(B)** Representative recording traces showing air-puff stimulation (60 ms; 60 psi)-evoked responses in a cerebellar PC and GL in treatments with ACSF, NA (15 μM), washout. **(C)** Bar graph showing the normalized amplitude of P1 during treatments with ACSF, NA, and washout. **(D)** Summary of data showing the normalized amplitude of N1 during treatments with ACSF, NA, and washout. ***P < 0.001 versus control (ACSF); n = 6 in each group.
